# Endobronchial ultrasound transbronchial biopsy with guide-sheath for the diagnosis of solitary pulmonary nodules

**DOI:** 10.18632/oncotarget.16813

**Published:** 2017-04-04

**Authors:** Chun-Hua Xu, Qi Yuan, Li-Ke Yu, Wei Wang, Yong Lin

**Affiliations:** ^1^ Endoscopic Center of Nanjing Chest Hospital, Nanjing, Jiangsu 210029, China; ^2^ Clinical Center of Nanjing Respiratory Diseases and Imaging, Nanjing, Jiangsu 210029, China; ^3^ Department of Respiratory Medicine, Nanjing Chest Hospital, Nanjing, Jiangsu 210029, China

**Keywords:** endobronchial ultrasound, transbronchial biopsy, guide-sheath, solitary pulmonary nodules

## Abstract

The aim of this study was to assess the usefulness of endobronchial ultrasound transbronchial biopsy with guide-sheath (EBUS-GS-TBB) for the diagnosis of solitary pulmonary nodules (SPNs). One hundred and eighty patients, who were diagnosed with SPNs and underwent an endobronchial ultrasound procedure. The diagnostic yield, safety and the associated factors were analyzed. Mean EBUS-GS procedure time was 14±8 min. The average number of biopsy specimens obtained in each SPNs was 5±1.2. One hundred and thirty-four SPNs were diagnosed by EBUS-GS-TBB and the diagnostic rate was 74.4 %. The diagnosis rate of malignancy was 83.3 %, while that of benign disease was 56.7 %. The most important factors that helped enhance EBUS-GS diagnostic accuracy included lesion diameter greater than 20mm, EBUS probe within the lesions and central lesions. No pneumothorax, hemoptysis or other serious complications occurred with the diagnostic procedures. EBUS-GS-TBB is a safe and effective method for diagnosing SPNs.

## INTRODUCTION

Bronchoscopy has been widely used in the evaluation of pulmonary lesions of the lung, however, the diagnostic yield of solitary pulmonary nodules (SPNs) is still low [1, 2]. Although the high diagnostic yield of computed tomography-guided percutaneous needle biopsy (CT-PNB) has been recognized, the complication rate of pneumothorax is nearly 20 % [3]. Endobronchial ultrasound (EBUS) extends vision beyond the airway walls to both peri-bronchial structures and distal peripheral lung lesions, guiding biopsies of pulmonary parenchymatous lesions [4]. EBUS images assisted transbronchial biopsy (TBB) has been performed in diagnosis pulmonary peripheral lesions [5]. Recent studies demonstrated that EBUS-TBB with guide sheath (GS) are useful for evaluating pulmonary peripheral lesions, with reported diagnostic rate of more than 70 % and low incidence of complication [6, 7]. However the role of EBUS-GS-TBB in the diagnosis of SPNs has not been fully examined. The present study was undertaken in order to examine the usefulness of EBUS-GS, as a guide for TBB and bronchial brushing cytology for diagnosis of SPNs.

## RESULTS

### Clinical characteristics

A total of 180 patients with SPNs were eligible for inclusion in this study (102 males and 78 females, mean age 58.5 years, ranged 33-76 years). One hundred and twenty patients were finally diagnosed of malignant diseases, including 106 cases of adenocarcinoma, 8 cases of squamous carcinoma, 2 cases of small cell carcinoma, 4 cases of lung metastatic nodule. Sixty patients were benign disease, of which pneumonia 18 cases, tuberculosis 20 cases, pulmonary abscess 2 cases, organizing pneumonia 8 cases, interstitial pneumonia 6 cases, pulmonary fungal infection 6 cases.

### Diagnosis of EBUS-GS-TBB

Duration of EBUS-GS time was 14±8 min, and the mean number of specimens obtained from each SPNs was 5±1.2. Ultrasound images were available in 156 lesions under EBUS guidance. The mean diameter of the lesion was 25±4.8 mm. Eighty-four lesions (46.7 %) were < 20mm and 96 lesions (53.5 %) were of 20-30 mm in mean diameter. The localization of the SPNs was the right upper lobe in 28 (15.6 %), right middle lobe in 24 (13.3 %), right lower lobe in 48 (26.7 %), left upper lobe in 30 (16.7 %) and left lower lobe in 50 (27.8 %).

One hundred and thirty-four of 180 patients were diagnosed by EBUS-GS-TBB, the diagnostic yield was 74.4 %, in which the diagnosis of malignant diseases was 83.3 % (100/120), benign diseases diagnostic yield was 56.7 % (34/60). One hundred malignant SPNs were composed of 92 adenocarcinomas, 4 squamous cell carcinomas, 2 small cell carcinomas and 2 metastatic carcinomas. For malignant lesions, the diagnosis of cytology and biopsy specimens was 66.0 % (66/100) and 76.0 % (76/100). Twenty false negative patients were confirmed after CT-PNB (12 cases of adenocarcinomas, 4 cases of squamous carcinomas, 2 cases of small cell carcinomas and 2 cases of pulmonary metastatic carcinomas). Thirty-four of the 60 benign lesions were diagnosed through EBUS-GS-TBB, the rest 26 lesions, 15 lesions were diagnosed by CT-PNB (1 case of pulmonary aspergillosis, 6 cases of pulmonary tuberculosis and 8 cases of pneumonia), and the other were diagnosed by surgical procedure (4 cases of organizing pneumonia, 1 case of pulmonary aspergillosis and 6 cases of pulmonary tuberculosis) (Table 1).

**Table 1:** Diagnosis of SPNs in 134 patients who underwent EBUS-GS-TBB

Lesions	Data
**Malignant**	
Adenocarcinoma	92
Squamous cell carcinoma	4
Small cell carcinoma	2
Metastatic carcinoma	2
**Benign**	
Tuberculosis	15
Organizing pneumonia	3
Pneumonia	11
Pulmonary abscess	1
Interstitial pneumonia	2
Pulmonary fungal infection	2

### Effect of EBUS-GS-TBB on diagnostic yield

**Figure 1:**
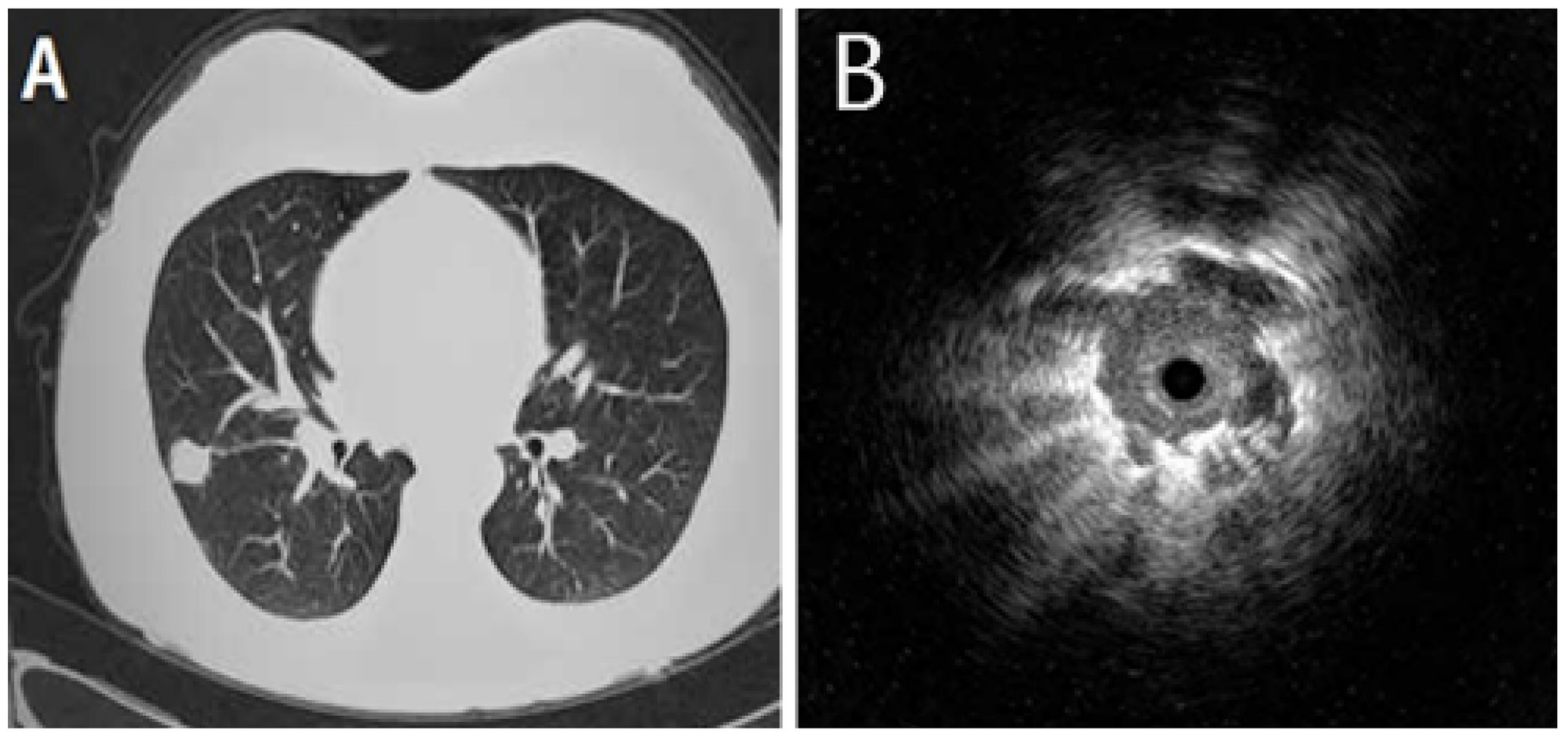
A 52-year-old male who underwent right middle lung lobectomy for pulmonary adenocarcinoma **(A)** Chest computed tomography showed a pulmonary nodule of 26 mm in diameter. **(B)** Endobronchial ultrasonography showed a low echoic nodule surrounded by a strong reflected interface produced between the aerated lung and the lesion.

We analyzed the influencing factors including size of the lesions, ultrasonography appearances and localization. Diagnostic yield of the SPNs in diameter of 20-30mm was 89.6 % (86/96), while the diameter of 20 mm or less was 57.1 % (48/84), the difference was statistically significant (χ^2^ = 12.39, *P* < 0.01). Lesion in which the probe was advanced to within the lesion, as determined from the EBUS image, had a higher diagnostic yield (102 of 110 lesions, 92.7 %) than when the probe was adjacent to the lesion on the EBUS image (32 of 46 lesions, 69.6 %) [χ^2^ = 7.18, *P* < 0.01] (Figure 1A). Diagnostic yield of the central lesions (medial 1/2 of lung field) on high resolution CT images was 83.3 % (100/120), while peripheral lesions (lateral 1/2 of lung field) was 56.6 % (34/60), the difference was statistically significant (χ^2^ = 7.48, *P* < 0.01) (Figure 1B and Table 2).

**Table 2:** Diagnostic yield for EBUS-GS-TBB

Variables	Lesion diagnosed by EBUS-GS-TBB	
	Malignant	Benign
Size of SPNs		
<20mm	32/84 (38.1%)	16/84 (19.0%)
20-30mm	68/96 (70.8%)	18/96 (18.8%)
Location of the probe		
Within	72/110 (65.5%)	30/110 (27.3%)
Adjacent	28/46 (60.9%)	4/46 (8.7%)
Location of the SPNs		
Medial 1/2 of lung field	76/120 (63.3%)	24/120 (20.0%)
Lateral 1/2 of lung field	24/60 (40.0%)	10/60 (16.7%)

### Complications

All patients tolerated EBUS-GS-TBB well. Self-limited bleeding was observed in 12 cases. Severe bleeding was not observed in this study. No pneumothorax, hemoptysis or other serious complications occurred with the diagnostic procedures.

## DISCUSSION

With the application of radial ultrasonic probe, EBUS can clearly show the lesions outside the lumen when reached the distal bronchus. EBUS with a radial probe is useful in sampling peripheral pulmonary lesions. Several studies have shown that EBUS-TBB is a safe and effective method for the diagnosis of pulmonary lesions [8, 9]. Therefore, EBUS-TBB was recommended for the diagnosis of lung lesions by the American College of Chest Physicians [10].

With the development of technology, GS has been introduced into EBUS-TBB. To evaluate the value of EBUS-GS-TBB in the diagnosis of peripheral pulmonary lesions, Kurimoto et al. carried out a research and found that the diagnostic yield was 77 %, there were only two patients with moderate bleeding, and no other complications occurred [7]. Kikuehi et al. identified that the EBUS-GS-TBB diagnostic rate was 58.3 % [6]. So far, there have been many researches showed that EBUS-GS-TBB is safe and effective in the diagnosis of pulmonary peripheral lesions [11–15].

EBUS-GS-TBB is useful in sampling peripheral pulmonary lesions. However, no trial has specifically addressed the yield in patients with SPNs. This is important information, as with the advent of increased CT imaging and screening, the number of small nodules detected is increasing.

In the present study, the diagnostic yield of EBUS-GS-TBB in SPNs was 74.4 %, and the diagnostic yield of benign and malignant diseases was 56.7 % and 83.3 %, respectively. Approximately 84.4 % of the lesions can be displayed on the EBUS, meanwhile, all lesions diagnosed by pathology were included in the detected 84.4 % lesions. With respect to the above, if the lesion could not be seen on the EBUS, the positive rate would be extremely low in the case of blind biopsy. Possible causes of the failure to detect the lesion by EBUS were listed as follows: lesions were located in the upper lobe of the lung, the probe sheath was difficult to reach the lesion due to the relationship between anatomical position and the structure of the trachea; the lesion was located near the pleural, too close to the peripheral region, the probe sheath was difficult to enter the terminal bronchiole, failed to cross the bronchus or close to the lesion, the probe was far from the lesion, thus unable to display.

Furthermore, the diagnostic yield of lesions with diameter larger than 20mm was obviously higher than that with the diameter less than 20mm, in accordance with findings indicated in the research performed by Yoshikawa et al and Yamada et al. [11, 16]. Accordingly, it is easier to get accurate pathological diagnosis of lesions with diameter over 20mm using EBUS-GS. Our investigation revealed that the diagnostic yield got under the situation that the probe within the lesion was higher than that the probe adjacent to the lesion, which was similar to the conclusion drawn from the study of Yamada et al [16]. Hence, the position of the probe should be adjusted following the probe reached the site of the lesion, so that the lesion can be completely wrapped around the probe as far as possible. Our investigation also indicated that lesions near the center from CT had an obviously higher diagnostic yield as compared to that near the peripheral. These results suggested that lesions in the paracentral regions with diameters over 20mm should have greater choice to be detected by EBUS-GS, and specimens should be obtained in the position that the lesion can be wrapped around the probe.

Compared with other diagnostic techniques, EBUS-GS maintained advantages. According to the results of several retrospective studies, the diagnostic yields of EBUS-GS-TBB and CT-PNB are similar, but pneumothorax occurred at lower frequencies in EBUS-GS-TBB [17, 18]. Another study reported that the diagnostic accuracy of CT-PNB was 95.2 % [19], higher with EBUS-GS-TBB in this study. A meta-analysis showed that the diagnostic yield of sheath guided TBB was slightly higher than virtual bronchoscopy and electromagnetic navigation bronchoscopy, but there was no significant difference [20]. With X-ray fluoroscopy, the diagnostic yield of EBUS-GS-TBB was about 67.1 %~77 %, and the yield was same with and without fluoroscopy demonstrated [13, 15]. Therefore, the diagnostic yield of EBUS-GS-TBB in peripheral pulmonary lesions is similar to other guided bronchoscopic techniques.

In conclusion, EBUS-GS-TBB is invasive, high diagnosing-rated and with few complications, used in the diagnosis of SPNs is safe and effective. At the same time, choosing appropriate patient is benefit to diagnostic accuracy.

## PATIENTS AND METHODS

### Patients

**Figure 2:**
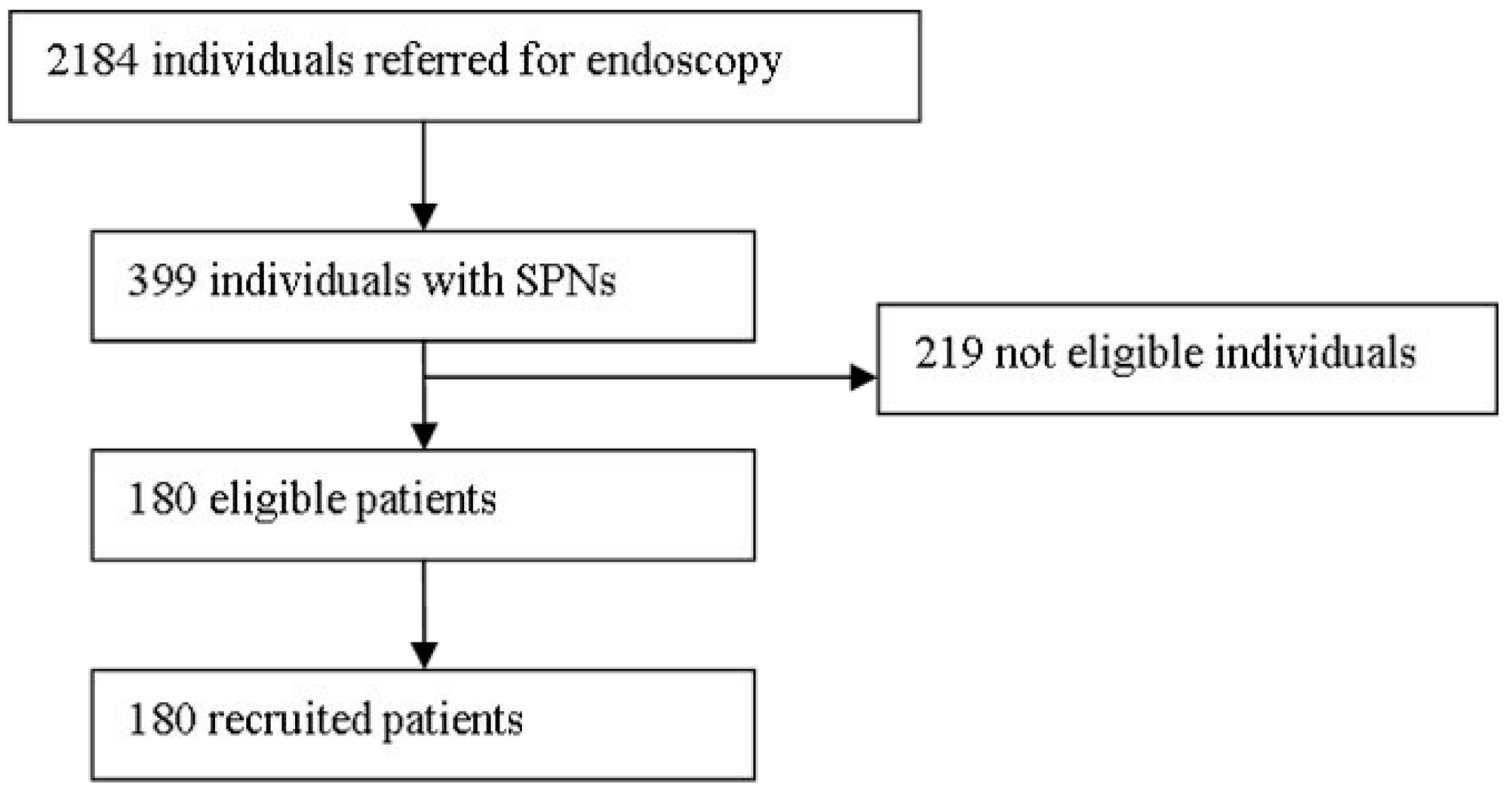
Flowchart of consecutive unselected patients referred to the endoscopy center

The study was designed to prospective evaluate the role of EBUS-GS-TBB in the evaluation of patients with SPNs. From January 2014 to August 2016, 2,184 consecutive unselected patients with suspected lung cancer were referred to Endoscopic Center of Nanjing Chest Hospital for diagnostic bronchoscopy. Patients with peripheral lung lesions were investigated by means of chest computed tomographs (CT). We screened 399 patients with SPNs, 219 patients were ineligible because they did not satisfy the inclusion criteria (Figure 2). One hundred and eighty patients with 180 SPNs ≤30 mm in mean diameter were enrolled for this study. SPNs were defined as those that were surrounded by pulmonary parenchyma and not visible by bronchoscopy.

For inclusion criteria, the characteristic of lung lesions accorded with the definition of SPNs, clinical and imaging data were visible, patients who agree to sign informed consent. Exclusion criteria were severe emphysema, multiple or single bullae in lung parenchyma near to SPNs lesion, cardio or pulmonary function insufficiency, hemorrhagic diseases or coagulation disorders, the patient underwent mental disorders or those can not cooperate the examination.

The study was approved by the Medical Ethics Committee of Nanjing Chest Hospital, Nanjing, China. All patients provided written informed consent before enrollment.

### EBUS-GS-TBB and bronchial brushing

The specific anatomical location of the lesions was reviewed by images before bronchoscopy. Bronchoscopy was performed through the nose route under local anesthesia with conscious sedation. Pre-medicated 6h solid food and liquid fasting and 2% lidocaine aerosol inhalation were routine procedure. Blood pressure, saturation of pulse oxygen and clinical symptom were recorded. Flexible bronchoscope of BF-1T260 or BF-1T240 type (Olympus, Japan) were used, EBUS was performed by an endoscopic ultrasound system (EU-M30S, Olympus, Japan), equipped with a 30-MHz mechanical radial-type probe (UM-S30-20R, Olympus, Japan), having an external diameter of 2.0mm, and guide sheaths (K-203, Olympus, Japan). A biopsy forceps or brush was inserted into GS before procedure adherence to guidelines, marked the position and then fixed probe into GS. The GS-covered probe was inserted through the work channel of the bronchoscope and advanced to the lesions to get the EBUS images. Adjust the probe to a suitable position where the lesions completely wrapped around the probe located. After obtaining the EBUS image, assistant helped to fix the bronchoscope and confirmed GS to entrance of working channel, then removed the probe and the GS remained in position. Biopsy forceps or brush acquired specimen via GS.

A bronchial brush was introduced into the sheath until the point marked by the cellulose tape reached the proximal end of the sheath. When using the brush, a few vigorous back-and-forth movements were performed to collect the sample on the brush under fluoroscopic guidance.

After the brushing forceps was withdrawn, the biopsy forceps was once again introduced into the sheath until the mark on the surface of the forceps reached the end of the sheath. Four to six biopsy specimens were obtained through the guide sheath using regular disposable biopsy forceps.

### Procedure of CT-PNB

The patients underwent CT in the prone, supine, or lateral position based on the shortest distance from the SPN lesion to the body chest surface. CT images were obtained from the region of interest by using a section thickness of 5 mm and were viewed by using lung window settings. After local anaesthesia with 2% lidocaine from the skin to pleura, a coaxial 18-gauge needle (Lot Number, REXK0682; Bard Peripheral Vascular, Inc., Tempe, AZ, 15 or 7cm in length) was inserted under intermittent CT guidance with its trajectory pointing toward the lung lesion. CT image comfirmed the tip of needle had entered the lesion, a cutting needle was penetrated into the lesion via the introducter trocar. This procedure was typically performed once, and occasionally performed twice. The patients were instructed to hold their breath during the CT scanning and the biopsy process. The resected specimen was placed in 10% formaldehyde for pathological examination. After removal of the biopsy needle, CT scanning was carried out to detect if any complication such as pneumothorax and haemorrhage occur, for necessary intervene.

### Time of procedure

The total time of EBUS-GS was defined as the time from the insertion of the probe to withdrawal of the guide sheath.

### Histological evaluation

If unable to obtain lesion images or biopsy specimens through EBUS, CT-PNB, following-up after treatment or surgery would be taken according to the patient's choice. And if the biopsy shows benign lesion, patient underwent follow-up after treatment. Two experienced pathologists read all the biopsy results, inconsistent results are determined by the discussion with the 3rd pathologist.

### Statistical analysis

Measurement data expressed by mean ± standard deviation, enumeration data expressed as a percentage. The analyses were carried out using SPSS 16.0 Statistics. χ^2^ test was used to compare the difference in percentages between groups. Value of *P*< 0.05 was considered statistically significant.
